# Sulfation of Birch Wood Microcrystalline Cellulose with Sulfamic Acid Using Ion-Exchange Resins as Catalysts

**DOI:** 10.3390/polym15051116

**Published:** 2023-02-23

**Authors:** Aleksandr S. Kazachenko, Natalia Yu. Vasilieva, Yaroslava D. Berezhnaya, Olga Yu. Fetisova, Valentina S. Borovkova, Yuriy N. Malyar, Irina G. Sudakova, Valentin V. Sychev, Noureddine Issaoui, Maxim A. Lutoshkin, Anton A. Karacharov

**Affiliations:** 1 School of Non-Ferrous Metals and Materials Science, Siberian Federal University, pr. Svobodny 79, Krasnoyarsk 660041, Russia; 2 Institute of Chemistry and Chemical Technology, Krasnoyarsk Scientific Center, Siberian Branch, Russian Academy of Sciences, Akademgorodok 50, bld. 24, Krasnoyarsk 660036, Russia; 3 Department of Biological Chemistry with Courses in Medical, Pharmaceutical and Toxicological Chemistry, Krasnoyarsk State Medical University, st. Partizan Zheleznyak, bld. 1, Krasnoyarsk 660022, Russia; 4 Laboratory of Quantum and Statistical Physics (LR18ES18), Faculty of Sciences, University of Monastir, Monastir 5079, Tunisia

**Keywords:** ion-exchange resin, cellulose, sulfamic acid, catalysis, sulfation

## Abstract

Cellulose sulfates are important biologically active substances with a wide range of useful properties. The development of new methods for the production of cellulose sulfates is an urgent task. In this work, we investigated ion-exchange resins as catalysts for the sulfation of cellulose with sulfamic acid. It has been shown that water-insoluble sulfated reaction products are formed in high yield in the presence of anion exchangers, while water-soluble products are formed in the presence of cation exchangers. The most effective catalyst is Amberlite IR 120. According to gel permeation chromatography, it was shown that the samples sulfated in the presence of the catalysts KU-2-8, Purolit s390 plus, and AN-31 SO_4_^2−^ underwent the greatest degradation. The molecular weight destribution profiles of these samples are noticeably shifted to the left towards low-molecular-weight compounds with an increase in fractions in the regions Mw ~2.100 g/mol and ~3.500 g/mol, indicating the growth of microcrystalline cellulose depolymerization products. The introduction of a sulfate group into the cellulose molecule is confirmed using FTIR spectroscopy by the appearance of absorption bands at 1245–1252 cm^−1^ and 800–809 cm^−1^, which correspond to the vibrations of the sulfate group. According to X-ray diffraction data, amorphization of the crystalline structure of cellulose is observed during sulfation. Thermal analysis has shown that with an increase in the content of sulfate groups in cellulose derivatives, thermal stability decreases.

## 1. Introduction

The main structural component of lignocellulosic biomass is cellulose. This is a valuable chemical raw material from which sought-after chemical compounds are obtained [1]. Paper, textile fibers, threads, varnishes, plastics, and hygiene products (such as tissue paper, tea towels, toilet paper) [2] are examples of the industrial uses of cellulose. The main approaches to cellulose processing are its depolymerization (including hydrolysis) to obtain valuable substances [3,4] and chemical modification [1,5,6].

Among the methods of chemical modification of cellulose, sulfation can be distinguished. Cellulose sulfation is one of the ways to obtain soluble sulfur-containing products with the required quality characteristics [7]. Cellulose sulfates can be used in various industries as thickeners, sorbents, ion-exchange materials in biotechnological and medical fields, etc. [8,9,10,11]. The biological activity of sulfated polysaccharides depends on their branching, chain length, and other physicochemical characteristics [12,13].

Most of the known methods of sulphation of cellulose [14,15,16,17], as well as of many carbohydrates and related compounds, and methods of selective sulfation of polysaccharides [18,19], are based on the use of three main reagents: sulfuric acid, sulfur trioxide, or its complexes with various basic reagents, and chlorosulfonic acid.

Traditional sulfation methods have a number of significant limitations due to the use of toxic and corrosive reagents. This imposes significant difficulties on the use of these methods in industry. However, a significant advantage of these methods is a high degree of sulfation and, accordingly, water solubility and bioavailability [14,15].

A mild sulfating agent with low toxicity, closely adjacent to sulfur trioxide complexes with bases, is sulfamic acid, which can be considered as a SO_3_–NH_3_ complex [16,17]. The process of sulfation with sulfamic acid is carried out in the presence of basic reagents [6,20], for example, urea. The main disadvantages of the sulfation process with sulfamic acid in the presence of bases are the insufficient knowledge of the role of urea in sulfation (although some screening of activators was studied earlier [21]) and the impossibility or difficulty of regenerating urea from the reaction mass.

Alternative methods of sulfation in ionic liquids [22] and deep eutectic solvents [23,24,25,26] are traditional methods. The advantage of these methods is the absence of organic solvents as a source of the medium and the possibility to exclude the use of toxic and corrosive reagents. The disadvantages include a high consumption of reagents as well as side reactions of carbamatization, the effects of which (including on bioactivity) have not been studied sufficiently.

Each of the listed methods of sulfation has both its advantages and disadvantages. Thus, traditional sulfation methods have a number of significant limitations due to the use of toxic and corrosive reagents.

The study of ion-exchange resins and other heterogeneous solid catalysts containing acidic or basic sites in various reactions has been carried out for more than half a century. Usually, they are used in the modification of low-molecular-weight compounds and their use is less common in the synthesis of popular low-molecular-weight products from natural polymers [27,28,29], alkylation, isomerization, aldolization, ketolization, ketalization and oligomerization of alkenes [30], etc. For example, they are used in the transformation of fructose into butyl levulinate [31], acylation, alkylation of aromatics, and Friedel–Crafts-related reactions [30].

It should be noted that the use of a heterogeneous catalyst eliminates the corrosive effect of acids on the metal, which simplifies the design of the process: the reaction products are easily separated from the catalyst by filtration [28], the washing of the catalyzate from the catalyst is facilitated, and the formation of a large volume of waste water is excluded. The absence of wastewater greatly simplifies the technological schemes.

Despite the fact that the processes of sulfation of natural organic substances have been developed for a long time, there is little information in the literature on the use of heterogeneous solid catalysts in this process. Weng et al. [14] used DMAP/DCC as a catalyst for the sulphation of cellulose with chlorosulfonic acid. Kazachenko et al. [32,33] studied solid catalysts with various acid sites (Brønsted and Lewis) during the sulfation of betulin with sulfamic acid. The work [34] describes the use of oxidized carbon supports and titanium and aluminum oxides as catalysts for sulfation of wheat straw lignin. Kazachenko et al. [34] showed that the most efficient catalyst is a modified carbon material with various acid groups. The development of catalytic sulfation methods can help solve the problem of catalyst recycling and reuse, and reduce the amount of reactants.

The mechanism of sulfation of polysaccharides by sulfamic acid is not well understood. However, it is assumed [16,35,36,37] that in order to effectively carry out the process of sulfation of natural hydroxyl-containing organic substances with sulfamic acid, it is necessary to weaken the S–N bond in sulfamic acid. The increase in the reactivity of sulfamic acid in the presence of the organic bases used as activators of sulfation of polysaccharides with sulfamic catalysts is explained by the formation of a donor–acceptor complex with a higher sulfation reactivity [16,35,36,37]. The rate of direct interaction of alcohols with sulfamic acid is lower than the rate of catalyzed sulfation, because the S–N bond in sulfamic acid is stronger than in the donor–acceptor complex [16,35,36,37]. An alternative mechanism was proposed for the catalytic effect of solid catalysts, which are Lewis or Brønsted acids, on the process of sulfation of lignin with sulfamic acid [34]. According to this mechanism, the zwitterionic form of sulfamic acid is first sorbed onto the catalyst matrix, which is a Brønsted acid, followed by its decomposition into sulfur trioxide and ammonia. The sulfur trioxide then reacts to form a reactive sulfating complex with 1,4-dioxane, which in turn sulfates the lignin molecule. Thus, during sulfation with sulfamic acid, the mechanism is possible with both basic and acid catalysts.

In this work, we studied the effect of heterogeneous anion-exchange and cation-exchange resin catalysts on the yield, properties, and composition of the reaction products in the microcrystalline cellulose sulfation process with sulfamic acid in 1,4-dioxane. Both strongly acidic and weakly acidic cation exchangers, as well as strongly basic and weakly basic anion exchangers, were chosen for the study.

The purpose of this work is to study the process of sulfation of microcrystalline cellulose with sulfamic acid in a medium of 1,4-dioxane in the presence of heterogeneous anion-exchange and cation-exchange resin catalysts, as well as to study the composition and structure of sulfation products using FTIR spectroscopy, X-ray diffraction, gel permeation chromatography, and elemental analysis.

## 2. Materials

The reagents used were aminosulfonic acid and 1,4-dioxane produced by Khimreaktivsnab (Ufa, Russia), KY-2-8 (OOO PO “TOKEM”, Kemerovo, Russia), Amberlite IR 120 catalyst produced by Sigma-Aldrich (Saint Louis, MO, USA), Purolite s390 plus produced by Purolite Co. (King of Prussia, PA, USA), and AP-3, ANKB-1, VP SO_4_^2−^, VP SO_4_^2−^, AB-17 Cl^−^, CG-1m kationit, and AN-31 SO_4_^2−^ provided by ICCT SB RAS.

### 2.1. Isolation of Microcrystalline Cellulose

The air-dried sawdust of birch wood (*Betula pendula*) with a fractional composition of 2–5 mm was used as the initial raw material. The content of wood components was determined by standard methods of chemical analysis [38]. The chemical composition of birch wood (% by weight of abs. dry wood) was as follows: cellulose 46.8, lignin 21.7, hemicellulose 27.3, extractives 3.9, and ash 0.3.

Birch sawdust was delignified in a glass reactor equipped with a mechanical stirrer and reflux condenser. The delignification solution was prepared using hydrogen peroxide (5 wt%) and acetic acid (25 wt%). The liquid/wood ratio (LWR) was 15. The delignification process was conducted at a temperature of 100 °C with permanent stirring for 4 h [39].

A cellulose product was obtained with a yield of 46.1 wt%, chemical composition (in wt%): cellulose 93.7, lignin 0.5, hemicellulose 5.5; crystallinity index 0.7; and degree of polymerization 456.

After sulfuric acid hydrolysis, the content of residual hemicelluloses decreased to 1.9 wt% in cellulose, the degree of polymerization decreased to 389, and the crystallinity index increased to 0.76.

### 2.2. Cellulose Sulfation

A total of 50 mL of 1,4-dioxane, 5 g of sulfamic acid, and 2 g (0.021 mol) of microcrystalline cellulose were added to a three-necked flask with a capacity of 250 mL. The mixture was heated to 90 °C with vigorous stirring, after which 0.5 g of the catalyst was added (the list is given in Table 1). The sulfation process was carried out for 3 h.

At the end of the process, the reaction mixture was cooled to room temperature, the solvent was decanted, the solid residue was dissolved in 25 mL of distilled water, neutralized with 5% ammonia solution until neutral, and filtered. An aqueous solution containing cellulose sulfate and reaction products was dialyzed against distilled water on an MF-503-46 MFPI cellophane dialyzing bag (USA) with a pore size of 3.5 kDa (−0.1 μm). The product was dialyzed for 10 h, during which the water was replaced every 1–2 h. After the dialysis process, an aqueous solution of the ammonium salt of cellulose sulfate was evaporated in a vacuum of a water jet pump at a temperature of 50–60 °C. The residue was transferred to a Petri dish and dried at a temperature of 50 °C to a constant weight. From the residue that did not dissolve in water, the catalyst was mechanically separated from the organic part. The yield and sulfur content in the organic residue were determined.

In a similar manner, sulfation was carried out without the use of ion-exchange resins as catalysts.

The yield of sulfated microcrystalline cellulose was calculated from the formula based on the sulfur content:

(1) $$Yield\left(\%\right)=\frac{m_{sc}\left(32-0.97\times S\right)}{32\times m}*100\%$$

where *m_sc_* is the mass obtained by sulfated MCC, g; *m* is the mass of the initial MCC; *S* is the sulfur content; 32 is the atomic mass of sulfur, g/mol; and 97 is the molecular weight of the ammonium sulfate group, g/mol.

*DS* is the degree of sulfation (the number of sulfate groups per one anhydride–glucose unit). It was calculated by the formula:

(2) $$DS=\frac{162\times S}{3200-97\times S\;}$$

where *S* is the sulfur content; 162 is the molecular weight of an anhydroglucose unit g/mol; 3200 is 100 atomic mass units of sulfur, g/mol; and 97 is the molecular weight of the ammonium sulfate group, g/mol. The degree of sulfation was 0 ≤ *DS* ≤ 3.

### 2.3. Analytical Methods

#### 2.3.1. Determining the Sulfur Content

The sulfur content in the sulfated cellulose was determined using a ThermoQuest FlashEA-1112 elemental analyzer (Waltham, MA, USA).

#### 2.3.2. Fourier Transform Infrared Spectroscopy

The FTIR spectra of the initial cellulose and sulfated cellulose were recorded on a Shimadzu IRTracer-100 FTIR spectrometer (Japan) in the wavelength range from 400 to 4000 cm^−1^. The spectral data were analyzed using the OPUS software (version 5.0). Solid specimens in the form of tablets in a KBr matrix (2-mg specimen/1000 mg of KBr) were prepared for the analysis.

#### 2.3.3. X-ray Diffraction

The XRD study was carried out on a DRON-3 X-ray diffractometer (Cu*K*α monochromatized radiation with λ = 0.154 nm) at a voltage of 30 kV and a current of 25 mA. The scanning step was 0.02 deg and the intervals were 1 s per data point. The measurements were performed in the Bragg angle (2θ) range from 5.00 to 70.00.

#### 2.3.4. Gel Permeation Chromatography

The determination of the degree of polymerization and molecular weight of initial MCC was determined by the method using an iron–vinyl–vanadium complex [40,41].

The weight-average molecular mass (Mw), number-average molecular mass (Mn), and polydispersity of the sulfated cellulose samples were determined by GPC using an Agilent 1260 Infinity II Multi-Detector GPC/SEC System chromatograph with two detectors: a refractometer (RI) and a viscometer (VS). The separation was achieved on two Agilent PL aquagel-OH columns using the aqueous solution of 0.2 M NaNO_3_ + 0.01 M NaH_2_ PO_4_ (pH 7) as a mobile phase. The column was calibrated using the polyethylene glycol standards (Agilent, Santa Clara, CA, USA). The eluent flow rate was 1 mL/min and the sample volume was 100 μL. Prior to the analysis, the samples were dissolved in the mobile phase (1–5 mg/mL) and filtered through a 0.22-μm Agilent PES membrane filter. The data were collected and processed using the Agilent GPC/SEC MDS software.

#### 2.3.5. Atomic Force Microscopy

The preparation of the sulfated cellulose films was carried out as follows: The sulfated cellulose (1 g) was dissolved in distilled water (30 mL) at room temperature. The resulting solution of sulfated cellulose was poured into a Petri dish and dried in an oven at a temperature of 45 °C to a constant weight. The obtained films of sulfated cellulose were separated from the Petri dish with tweezers, after which they were analyzed by atomic force microscopy. The study of the sulfated cellulose films by AFM in a semicontact mode was carried out using a Solver P47 multimode scanning probe microscope (NT-MDT, Moscow, Russia). Scanning was performed at no less than 3–4 points at several sites. Scan speed was 1.5–2.0 Hz and the resolution of the resulting image was 256 × 256 pixels.

#### 2.3.6. Thermal Analysis

The thermogravimetric study and data analysis were performed using a NETZSCH STA 449 F1 Jupiter simultaneous thermal analysis instrument (Selb, Germany). The thermal degradation of the samples was analyzed in argon in the temperature range from 30 to 800 °C; the protective and purge gas flow rates were 20 and 50 mL/min, respectively. The samples were heated in a dynamic temperature regime (10 °C/min) in corundum crucibles. The measurement results were processed using the NETZSCH Proteus Thermal Analysis 5.1.0 software supplied with the instrument.

#### 2.3.7. Ultraviolet–Visible Diffuse Reflectance Spectral (UV-DR) Analysis

Ultraviolet–visible diffuse reflectance spectra (UV-DR) were measured with the UV-Shimadzu 3600 scanning spectrophotometer using an ISR-603 integrating sphere attachment. The baseline was recorded using high-purity BaSO_4_.

## 3. Results and Discussions

### 3.1. Catalytic Synthesis of Cellulose Sulfates

Cellulose sulfate (CS) has been known for over 100 years, but the sulfation process is still not well understood. One reason is that cellulose sulfate in its acidic form is unstable and easily breaks down further into low-molecular-weight products. In addition, the synthesis of CS is complex; the sulfation reaction is accompanied by the processes of depolymerization of both the original cellulose and its sulfation products. It is rather problematic to control these reactions [42].

When choosing the most rational sulfation method for the production of cellulose sulfate, which has a set of properties that are important for its use in specific areas [8,9,43,44,45], it is necessary to take into account a combination of a number of factors. The main factors which should be considered are not only the simplicity of the equipment design, degree of conversion, yield, and degree of substitution of the sulfated product, but also the structure and molecular weight of the product [6], and the possibility of recycling and reuse of the catalyst [33].

Data on the effects of various types of ion-exchange resins on the sulfur content, yield, molecular weight, and polydispersity of samples of the obtained cellulose sulfates are shown in Table 1.

**Table 1:** Influence of the catalyst on the yield, sulfur content, and molecular weight of cellulose sulfate during sulfation with sulfamic acid.

№	Catalyst *	Sulfur Content in Water-Soluble Sulfate, wt. %	DS	Yield of SC, %		Properties of Ion Exchangers	Mw	PD
				+H_2_O	−H_2_O **			
0	-	7.60	0.50	trace	85.10	-	-	-
1	Amberlite IR 120	19.60	2.44	45.30	11.30	Strong acid cation exchanger (H-form) gel type sulfonated polystyrene type.	6708	2.256
2	KY-2-8	20.50	2.74	25.90	7.20	Strong acid cation exchanger.	3146	1.613
3	CG-1m kationit	15.90	1.55	12.90	22.30	Weakly acidic macroporous cation-exchange resin with a polyacrylic matrix containing functional carboxyl groups. Completely aliphatic matrix, polymethacrylic acid chains are linked by triethylene glycol dimethacrylate.	8257	2.388
4	Purolit s390 plus	20.10	2.60	18.30	8.90	Weakly acidic macroporous cation exchanger with iminodiacetic acid chelate groups.	3325	1.552
5	ANKB-1H	16.00	1.57	5.70	75.80	Polyampholyte pyridine, COOH.	8353	2.357
6	AN-31 SO_4_^2−^	19.40	2.38	16.40	41.10	Low-basic amino-functional anion exchangers, a polymeric combination of divinylbenzene with styrene. Structure three-dimensional, gel, macroporous.	4181	1.932
7	AP-3	16.30	1.63	7.40	61.60	Strong base anion exchanger, bifunctional. Macroporous structure.	7480	2.254
8	VP SO_4_^2−^	16.80	1.73	4.30	78.10	Strongly basic macroporous anion-exchange resin based on a copolymer of 4-vinylpyridine and divinylbenzene.	9756	2.768
9	AB-17 Cl^−^	17.40	1.86	8.90	60.10	Anion-exchange resin strongly basic ion-exchange with a gel structure	5536	2.229

* Cation exchanger resins were in the H-form. ** The sulfur content in water-insoluble products was 2.5–3.5% (mass.).

It is evident from the data in Table 1 that the use of ion-exchange resins in the sulfation process led to the formation of products with a high degree of sulfation (1.55–2.74). The maximum degree of sulfation of 2.74 sulfated product was obtained with a strongly acidic cation exchanger (KU-2-8). At the same time, the yield of water-soluble sulfated products was no more than 45.3% and was also obtained with a strongly acidic cation exchanger. Simultaneously with the process of sulphation of cellulose with sulfamic acid in the presence of all catalysts, the process of destruction of the initial polymer occurs, which is primarily a consequence of the action of sulfamic acid, which is enhanced by the presence of cation exchangers. Sulfation with anion exchangers is mainly accompanied by a lower degree of destruction of the polysaccharide and higher yields of water-insoluble sulfates. With an increase in the degree of degradation, an increase in the degree of sulfation of the product is observed, which may be due to the greater availability of hydroxyl groups of a low-molecular-weight polymer for attack by a sulfating reagent. At the same time, a decrease in the yield of water-insoluble sulfate is observed. It should be noted that the yield of water-soluble sulfates is significantly higher when using strong cation exchangers. It can be assumed that under these conditions the mechanism proposed by the authors of [20] is implemented.

Thus, water-insoluble sulfated reaction products are formed in high yield in the presence of anion exchangers, while water-soluble products are formed in the presence of cation exchangers. Without the use of catalysts, only traces of water-soluble and about 80% water-insoluble sulfates are formed.

### 3.2. Gel Permeation Chromatography of Sulfated Cellulose

**Figure 1:**
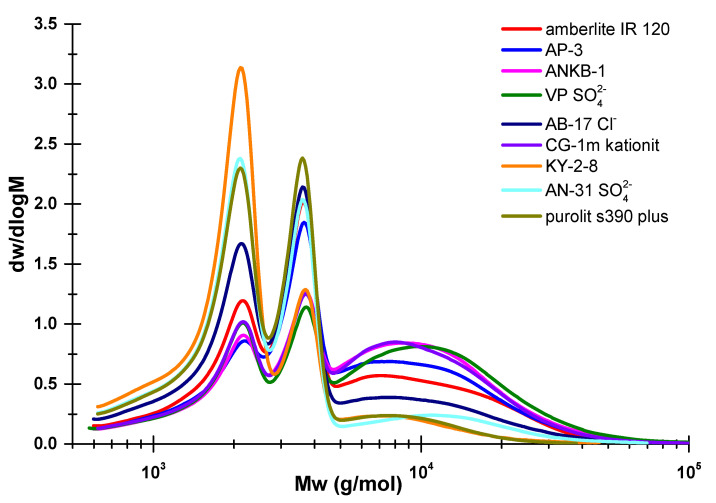
Molecular weight distribution of cellulose sulfates obtained with various catalysts.

The molecular weight distribution (MWD) data presented in Figure 1 show that during the sulfation of MCC extracted from birch wood, in the presence of all catalysts, the cellulosic polymer structure changes. All obtained samples have a rather low average molecular weight (Mw) (up to 9800 g/mol) compared to the initial MCC (~46,000 g/mol), as well as trimodal MWD with polydispersity index (PDI) values not exceeding 2.8. However, it should be noted that the samples sulfated in the presence of the catalysts KU-2-8, Purolit s390 plus, and AN-31 SO42- underwent the greatest degradation. The MWD profiles of these samples are noticeably shifted to the left towards low-molecular-weight compounds with an increase in fractions in the regions Mw ~2100 g/mol and ~3500 g/mol, indicating the growth of MCC depolymerization products. The lowest PDI values of the samples (1.5–1.9) obtained in the presence of the KU-2-8, Purolit s390 plus, and AN-31 SO_4_^2−^ catalysts are associated with a small amount of macromolecular compounds in the structure and the predominant content of polymer particles with Mw below 5.000 g/mol, which makes them more uniform in structure compared to samples modified in the presence of other catalysts.

### 3.3. FTIR

**Figure 2:**
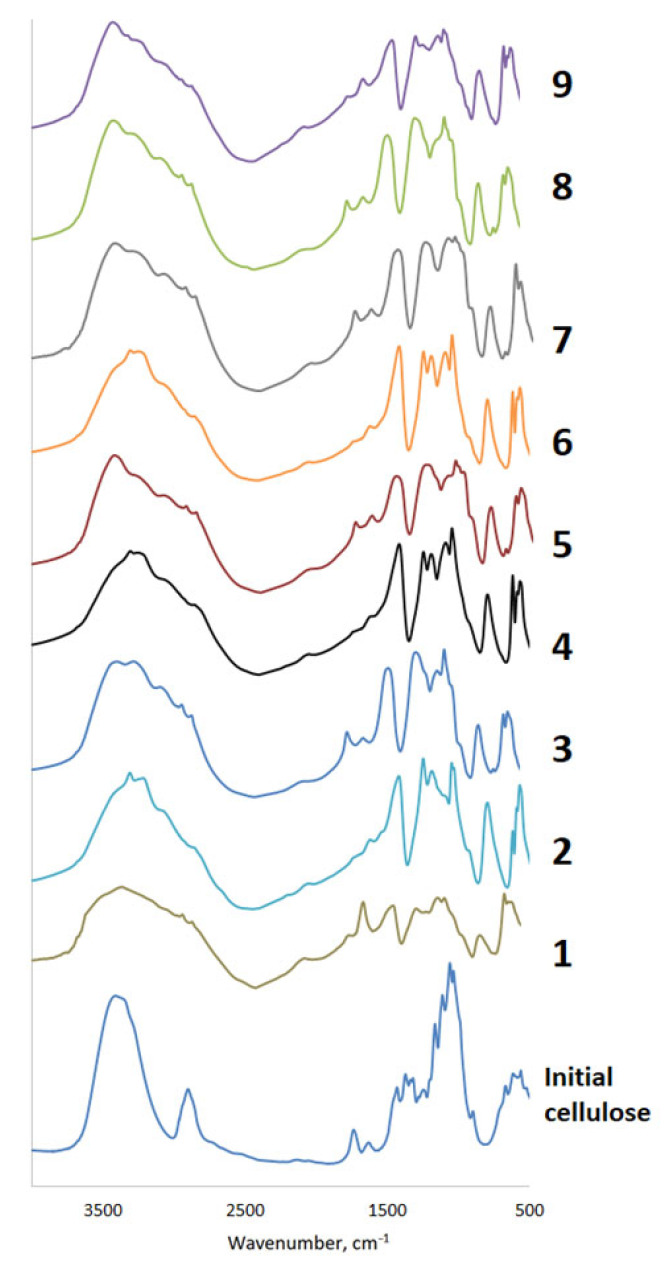
FTIR spectra of cellulose and sulfated cellulose samples (the numbers alongside the FTIR spectra of cellulose sulfates correspond to the studied catalyst (Table 1)).

Cellulose sulfates obtained using various catalysts were analyzed using FTIR spectroscopy (Figure 2).

The results of FTIR spectroscopy confirm the introduction of a sulfate group into the MCC molecule. In the FTIR spectra of cellulose sulfates, in comparison with the original cellulose, there are absorption bands in the region of 1245–1252 cm^−1^ and 800–809 cm^−1^, which correspond to the vibrations of the sulfate group. The intensities of the two new peaks increase with the DS. There is also a broadening of the absorption bands in the region of 2800–3500 cm^−1^, caused by the superposition of the absorption bands of the hydroxyl and ammonium alkyl groups. It should be noted that the main shapes of the spectra of cellulose sulfates obtained by different methods are the same. In the IR spectra of sulfated samples, compared with the spectra of the original MCC, there is a decrease in the intensity of the absorption band of stretching vibrations of OH groups and a shift in the absorption band of stretching vibrations of OH groups in the region of 3410–3405 cm^−1^ to the high-frequency region, the region of 3510–3500 cm^−1^, which is explained by a decrease in the number of hydrogen bonds in the sulfated MCC compared to the original MCC.

The results obtained are consistent with the data presented in the works [23,46].

### 3.4. X-ray Diffraction

**Figure 3:**
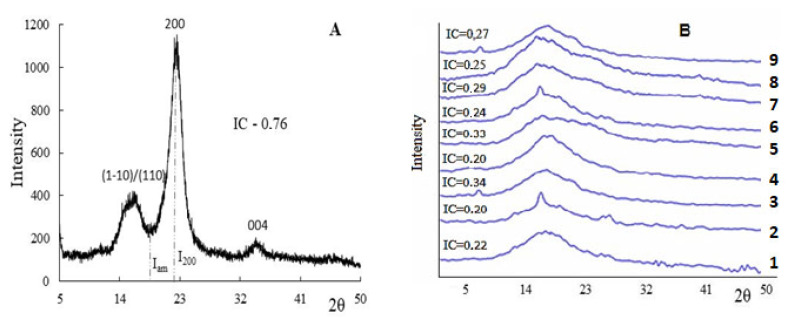
XRD patterns of (**A**) cellulose and (**B**) sulfated cellulose samples (the numbers of the diffraction patterns correspond to the studied catalyst (Table 1)).

Cellulose sulfates obtained using various catalysts and the initial MCC were studied by XRD (Figure 3).

Based on the concept of the structure of microcrystalline cellulose as a polymer consisting of crystalline and amorphous phases, an analysis of diffraction patterns was carried out. According to the data shown in Figure 3, the original birch microcrystalline cellulose has two characteristic peaks at around 14.7°, 16.4°, 22.4°, and 34.5°, corresponding to the crystal faces (1ī0), (110), (200), and (004), respectively, which correspond to the reflection of the monoclinic phase of cellulose I [47]. The crystallinity index measured on the ratio of intensities at two points of diffractograms (according to Segal) [47,48] is 0.86.

In the process of sulfation, amorphization of the initial structure of microcrystalline cellulose is observed, the peaks are smoothed in the region of 12–35° 2θ. The observed phenomenon corresponds to the data presented in the literature, when the chemical modification of polysaccharides reduces their crystallinity and increases amorphization [6,49,50,51].

### 3.5. Thermal Analysis

**Figure 4:**
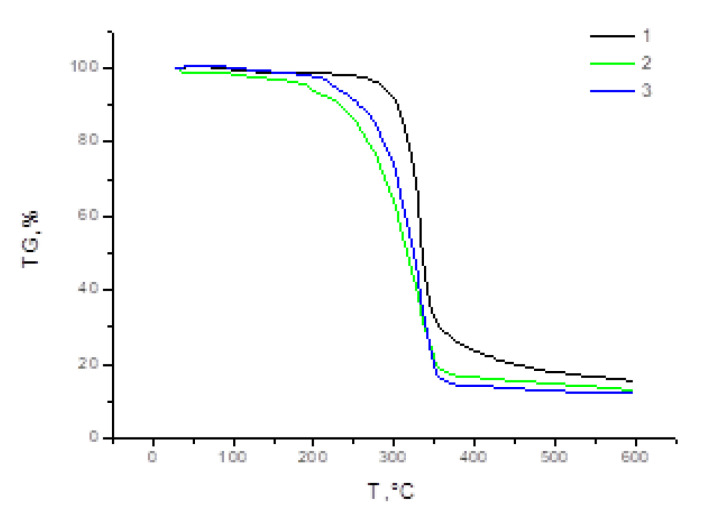
Thermogravimetric curves of cellulose (1) and its sulfated derivatives obtained with Amberlite IR 120 (2) and AB-17 Cl^−^ (3) catalysts.

Figure 4 shows the thermogravimetry curves of the original cellulose and its sulfated modifications. Weight loss at temperatures below 100 °C is due to the evaporation of physically bound water. After that, the original cellulose practically does not lose weight up to 268 °C. On further heating, there is a rapid weight loss between 300 and 360 °C, followed by a slow weight loss up to 600 °C. This is a typical picture of the thermogravimetric process of cellulose pyrolysis [52].

The nature of the thermal decomposition of samples of cellulose sulfates obtained with the catalysts Amberlite IR 120 and AB-17 Cl^−^ differs from the thermolysis of cellulose. The temperature range of the dehydration process of these samples is much smaller, which is due to the increased content of low-molecular-weight fractions in the modified cellulose, from which the removal of chemically bound water occurs much faster than from the original cellulose [53].

The thermogravimetric curve of the cellulose sulphate sample prepared with the Amberlite IR 120 catalyst shows a slight jump in weight loss at 190 °C, and for the cellulose sulphate prepared with the AB-17 Cl^−^ catalyst, at 213 °C. This behavior may be associated with the degradation of highly sulfated areas of the cellulose structure. It is assumed that the presence of sulfate groups on the surface catalyzes the process of thermal decomposition, resulting in a region with less resistance to pyrolysis [54,55]. The higher temperature process may relate to the thermal decomposition of unsulfated regions of cellulose structure [56].

**Figure 5:**
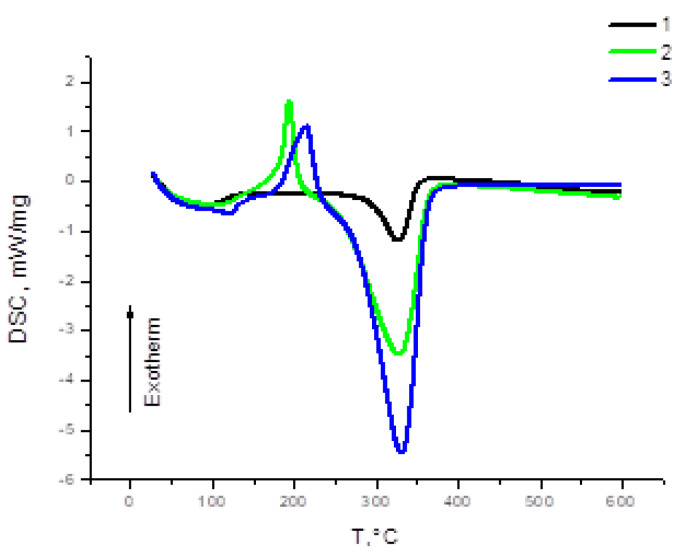
DSC curves of cellulose (1) and its sulfated derivatives obtained with Amberlite IR 120 (2) and AB-17 Cl^−^ (3) catalysts.

Figure 5 is a graphical interpretation of differential scanning calorimetry (DSC) data. For the studied samples, endothermic peaks below 110 °C are associated with the removal of liquid and sorbed water. The DSC curves of samples 2 and 3 show intense exothermic peaks with maxima at 193 and 213 °C, respectively, passing into the endothermic zone with an increase in the heating temperature. The endothermic effect in this area is associated with the decomposition of the structure of the substances. The latter statement is confirmed by the slope of the TG curve in this interval.

Then, the active decomposition of the structure of cellulose sulfates obtained with the Amberlite IR 120 and AB-17 Cl^−^ catalysts began; the temperature at the beginning of the active decomposition phase was 203 and 223 °C, respectively. This stage corresponds to a sharp weight loss on the TG curve up to a temperature of 358 °C. As is known [57], the temperature at the beginning of the main decomposition characterizes the thermal stability of a substance. Thus, the most thermally stable sample of cellulose sulfate was obtained with the catalyst AB-17 Cl^−^, which contains less sulfo groups. It can be assumed that an increase in the amount of sulfur can contribute to a decrease in thermal stability, since the thermal stability of the sulfoether bond is lower than that of bonds in the polysaccharide [58]. Further heating of the samples to 600 °C lead to the aromatization of the structure with the formation of a carbonized residue.

### 3.6. AFM

**Figure 6:**
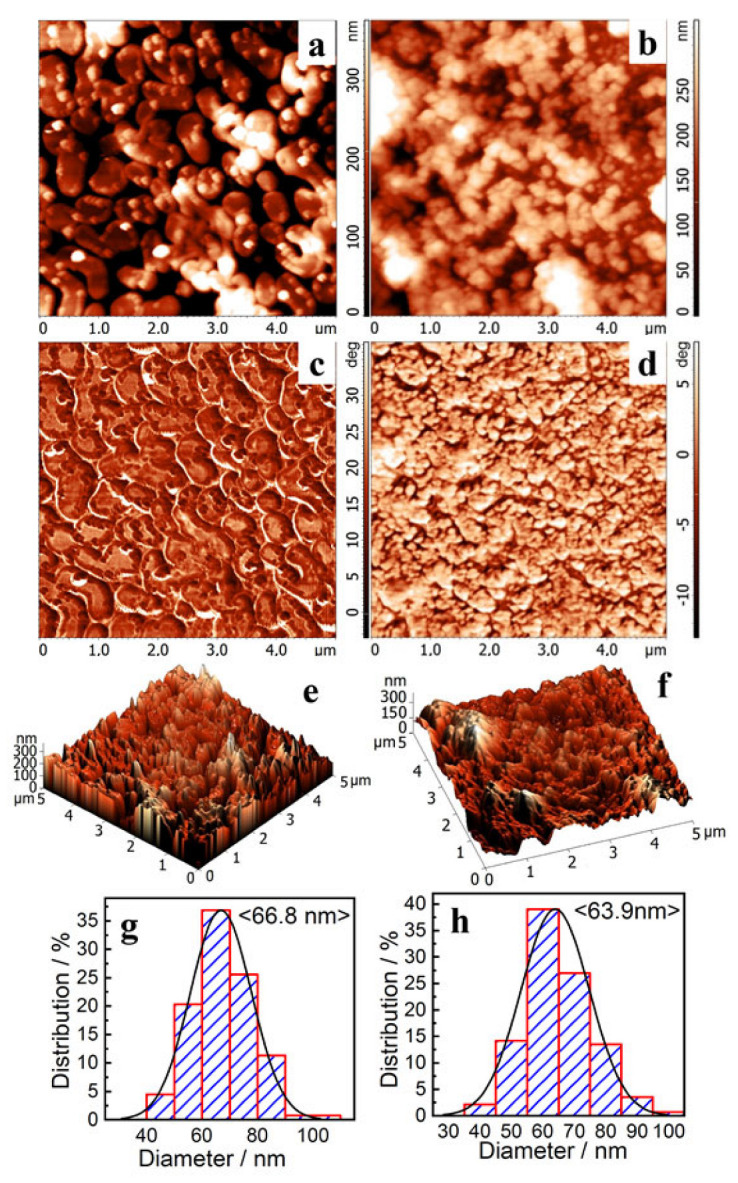
AFM data: 2D (**a**,**b**) and 3D (**e**,**f**) relief and phase contrast (**c**,**d**), as well as particle size distribution histograms (**g**,**h**), for samples of Amberlite IR 120 (**a**,**c**,**e**,**g**) and AB-17 Cl (**b**,**d**,**f**,**h**) catalysts.

The surfaces of the cellulose sulfate films obtained with the Amberlite IR 120 and AB-17 Cl^−^ catalysts were studied by atomic force microscopy (Figure 6).

According to atomic force microscopy data (Figure 6), the surface of the cellulose sulfate films obtained with the Amberlite IR 120 and AB-17 Cl^−^ catalysts consists of particles of various shapes and sizes, partially assembled into agglomerates. According to the phase contrast, no foreign inclusions were found.

Histograms for both studied samples have a normal Gaussian distribution. The average particle sizes for cellulose sulfate made with Amberlite IR 120 and AB-17 Cl^−^ catalysts are 66.8 nm and 63.9 nm, respectively.

### 3.7. Ultraviolet–Visible Diffuse Reflectance

**Figure 7:**
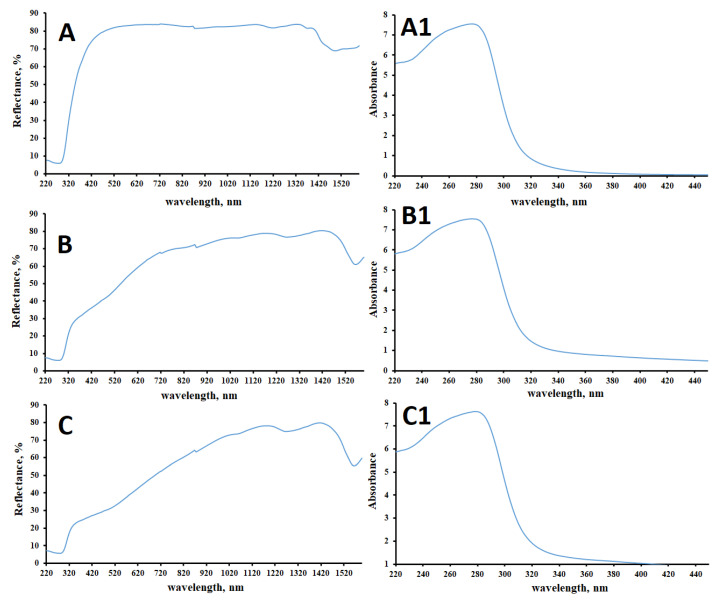
DR-UV spectra (**A**–**C**) and normalized Kubelka–Munk function F(R_∞_) (**A1**–**C1**) of initial cellulose (**A**,**A1**), sulfated cellulose samples of Amberlite IR 120 (**B**,**B1**), and AB-17 Cl (**C**,**C1**) catalysts.

All samples have the transition within 220–520 nm. As can be seen from Figure 7, the valence band boundary of the sulfated cellulose samples is shifted to the region of 310 nm (4.00 eV), while for the original cellulose this transition is observed in the region of 338 nm (3.67 eV) [59]. This indicates that the presence of the sulfate group narrows the boundaries between the valence band and the conduction band. This may be due to the additional degrees of freedom of electrons that provide the energy levels of sulfur and additional oxygen atoms in the electron phase space. The Kubelka–Munk function (Figure 7A1–C1) demonstrates that the maximum absorption of the studied samples is not greatly affected by the presence of a sulfate group.

## 4. Conclusions

The effect of ion-exchange resins as catalysts on the sulfation of cellulose with sulfamic acid was studied for the first time. It has been shown that water-insoluble sulfated reaction products are formed in high yield in the presence of anion exchangers, while water-soluble products are formed in the presence of cation exchangers. Sulfation of cellulose with an Amberlite IR 120 catalyst leads to the production of water-soluble cellulose sulfate with a yield of up to 45% and a sulfur content of up to 19.6 wt%. The introduction of the sulfate group was confirmed by elemental analysis and IR spectroscopy. The influence of the introduction of a sulfate group on the crystallinity, thermal stability, and morphology of the films is shown. The original cellulose and its sulfated derivatives were also examined by DR-UV. It is shown that the valence band boundary of sulfated cellulose samples is shifted to the region of 310 nm (4.00 eV), while in the original cellulose this transition is observed in the region of 338 nm (3.67 eV), which indicates that the presence of the sulfate group narrows the boundaries between the valence band and the conduction band.

A further prospect for this research may be the development of efficient heterogeneous catalysts for the sulfation of polysaccharides with sulfamic acid to provide both a high yield of water-soluble products and a high sulfur content.

## Data Availability

The data that support the findings of this study are available on request from the corresponding author.
